# Utilization of screening mammography in older women according to comorbidity and age: protocol for a systematic review

**DOI:** 10.1186/s13643-016-0345-y

**Published:** 2016-10-04

**Authors:** Joshua Demb, Isabel Allen, Dejana Braithwaite

**Affiliations:** 1 Department of Epidemiology and Biostatistics, University of California, 550 16th Street, 2nd Floor, Box 0560, San Francisco, CA 94143 USA; 2 Helen Diller Family Comprehensive Cancer Center, University of California, San Francisco, CA USA

**Keywords:** Screening mammography, Comorbidity, Functional limitations, Health status, Aging, Systematic review

## Abstract

**Background:**

Approximately half of new invasive breast cancer cases diagnosed each year in the United States occur among women aged 65 years and older. The increasing life expectancy coupled with the attendant rise in breast cancer incidence and elimination of out-of-pocket expenses for screening mammography as a result of the Affordable Care Act could lead to higher utilization rates of screening mammography. Although research indicates that life expectancy should be a strong consideration when making screening decisions among older women, the extent to which screening mammography utilization is tailored to comorbidity and life expectancy is not well established.

**Methods/design:**

To identify relevant studies, a systematic search of the literature will be conducted using PubMed and EMBASE between January 1, 1991, and March 1, 2016. Additional studies will be found through citation review or by contacting experts in the field. The inclusion criteria will be any study design comprised of women aged 65 and older, assessing women’s comorbidity, functional impairments, and/or health status, and reporting outcome measures that addressed mammography utilization within the last 1–5 years. For each study, two authors will independently abstract data regarding study eligibility and outcomes to determine relevance. Quantitative results will be extracted from text and tables, choosing preferably those adjusted for important confounders.

**Discussion:**

The review will provide evidence on the impact of comorbidity, functional limitations, and health status on screening mammography utilization in older women and inform decision aids in this area.

**Systematic review registration:**

PROSPERO CRD42016032661

**Electronic supplementary material:**

The online version of this article (doi:10.1186/s13643-016-0345-y) contains supplementary material, which is available to authorized users.

## Background

About half of new invasive breast cancer cases diagnosed each year in the United States (US) occur among women aged 65 years and older [1, 2]. The median age of breast cancer diagnosis is 61 years of age, with a median age at death of 68 years [3]. According to the US Preventive Services Task Force (USPSTF), insufficient evidence is available to recommend screening in women over age 74 years [4]. In 2015, the American Cancer Society (ACS) recommended continued screening for women aged 70 years and older on a routine basis as long as they are in good health [2, 5].

The incidence of breast cancer, the second most common cause of cancer death in US women, increases with age [6]. Recently, approximately 65–70 % of women aged ≥70 years continuously reported undergoing screening mammography from 2007 to 2012 in the Behavioral Risk Factor Surveillance System [7]. Consistent with this, data from the National Health Interview Survey [8] show considerable screening utilization among women in poor health. Thus, many older women undergoing screening mammography experience the consequences of screening without knowing if there is a benefit from screening. Given the long natural history of breast cancer in older women and increased competing causes of death, there comes a time when older women may not live long enough to benefit from screening mammography [9–11]. A recent meta-analysis concluded that breast cancer screening is most appropriate in women with a life expectancy of at least 10 years [2, 12, 13].

To date, there has been no published synthesis of evidence on screening mammography utilization in older women in relation to comorbidity or functional limitations. Comorbidity is generally evaluated based on the number and severity of individual conditions. Two notable comorbidity indices include the Charlson Comorbidity Index, which estimates the risk of mortality from comorbid disease by summing and weighting each condition, and the Elixhauser Index, which assesses patient comorbidity based on ICD-9-CM and ICD-10 codes found in administrative data [14–16]. Functional limitations are generally examined based on activities of daily living (ADLs), which are routine activities that individuals do every day without needing assistance, including eating, bathing, dressing, toileting, walking, and continence [17]. Functioning can also be ascertained using instrumental activities of daily living (IADLs), defined as complex skills necessary to live independently, such as managing finances, preparing meals, and managing medications and basic home maintenance [18]. Scales include ADLs or IADLs or a combination of the two to derive a common score.

### Objectives

In this review, we will synthesize the current evidence on screening mammography utilization in older US women as a function of comorbid conditions, functional limitations, and perceived health status. To address this clinical and policy conundrum, it is important to determine the extent to which life expectancy affects screening mammography utilization. We hypothesize that screening mammography utilization may vary according to advancing age and life expectancy [19, 20].

## Methods

We consulted the Preferred Reporting Items for Systematic Review and Meta-Analysis Protocols (PRISMA-P) guidelines while preparing this protocol and included them as Additional file 1 [21].

### Eligibility criteria

The broad inclusion criteria for this review allow for any study design published in English, including decision analyses, that (i) include women aged 65 years and older in the US, (ii) assess women’s comorbidity (either as a specific condition or a summary score) and/or functional impairments and/or health status, and (iii) report an outcome measure that addresses recent screening mammography utilization. We plan to include any randomized clinical trials and cohort, case–control, quasi-experimental, and cross-sectional studies that meet the above inclusion criteria. Case reports will be excluded. Most, if not all, of the target population are Medicare beneficiaries, with screening mammography covered based on policy changes implemented in 1991 [22]. Women in this age range are near the upper limit of the USPSTF primary screening mammography guidelines (age 74 years) and include age groups where data are currently inconclusive to provide screening recommendations [9]. To account for the Medicare policy effect, we will exclude studies evaluating screening utilization prior to 1991 [22]. Studies must either have complete populations aged 65 years and older or specific subgroups aged 65 years or older to be included in the review.

### Information sources and search strategy

The following electronic health databases will be searched: MEDLINE (using the PubMed interface), Excerpta Medica Database (EMBASE), Cumulative Index to Nursing and Allied Health Literature (CINAHL), and PsycINFO. A systematic search of the literature will be conducted between January 1, 1991, and March 1, 2016, to identify all relevant studies in English. Additional studies will be found by searching editorials, letters, overview-type articles, and citations of review articles or by contacting experts in the field regarding any unpublished articles that might be suitable for inclusion.

A search strategy to identify relevant studies will be developed in collaboration with a librarian. We will derive initial terms from Medical Subject Headings (MeSH) of key articles and from other relevant reviews. We will use “breast neoplasms” combined with permutations, variations, and abbreviations of the relevant MeSH keywords and non-MeSH key terms for screening mammography, age and comorbidity, functional limitations, and health status. Comorbidity terms will include Charlson and Elixhauser conditions and any other study comorbidity. An example of a search used for this review is included in the “Search strategy” section of the Appendix.

### Outcome of interest

The outcome is the screening mammography utilization in women, defined as screening mammography that occurred within the last 1–5 years. We anticipate that studies will measure screening mammography utilization within the previous 1–5 years and want to ensure that our review will exclude studies that simply measure ever having received screening mammography, since it does not adequately represent recent utilization.

### Data management and study selection process

All articles from our MEDLINE and EMBASE searches will be combined, and duplicates will be removed. As a primary screening, titles and abstracts will be reviewed against the eligibility criteria by two members of the review team (JD and DB) in duplicate, who both have expertise in cancer epidemiology and screening. When an article passes primary screening for either member of the review team, the full-text version of the study will be obtained and imported into Mendeley Web (bibliographic database) for easy access among review team members. Reports of the same study will be grouped together for easier data extraction. Using a pre-determined database, we will assess eligibility of each study by reading the full text. The form will specify all of the eligibility criteria and consist of a table with the total list of full-text studies retrieved for our records. Along with these criteria, reviewers will assess the quality of the studies on the same form.

Comments will be included for excluded studies to explain the reasoning behind exclusion. The final list of studies to be included in the review will be added to a database for further data extraction. A PRISMA flowchart will be completed to summarize the entire selection process.

### Data extraction

A data extraction form will be used to collect all of the relevant information from the selected studies. The form will include information about study characteristics, such as the type of study, number of participants, length of follow-up, outcome, and quality assessment. Exposures logged in this form will separately include comorbidity scales or specific diseases considered, functional limitation scales used, and measures of health status. The primary outcome is screening mammography utilization, defined as screening mammography that occurred within the last 1–5 years; odds ratios and the corresponding 95 % confidence intervals will serve as our effect estimates, with some studies also showing proportions of utilization. Summary measures from study participants such as measures of socioeconomic status, including education and income, health insurance, and number of physician visits will be extracted. One team member (JD) will extract the data from all studies. One other reviewer will independently assess the data extraction of the other team member for quality assurance. Any discrepancies will be discussed and resolved by the review team.

### Quality assessment

The team member responsible for data extraction (JD) will also conduct the quality assessment, which will be reviewed by a second team member (DB). As part of the data collection process, we will use the Newcastle-Ottawa Scale to measure the methodological quality of the selected observational studies [23]. Additionally, the Cochrane Collaboration Risk of Bias tool will be used to assess the quality of randomized controlled trials [24]. These scales will provide a summary of the quality ratings of the included studies for descriptive purposes only.

### Data synthesis

#### Narrative synthesis

We will conduct a narrative synthesis to describe the findings of included studies, explore associations of interest, and examine the quality of the studies and robustness of the systematic review. Study characteristics, including effect estimates and main findings, will be summarized comprehensively. We will tabulate the full Newcastle-Ottawa and Cochrane risk of bias findings of individual studies for descriptive purposes (see Additional file 2).

#### Quantitative synthesis

Results from each study will be compiled in summary tables for descriptive comparisons of study findings (see Additional file 3). We will evaluate three exposures: comorbidity, functional limitations, and health status. For each exposure, we will aggregate study findings to perform meta-analyses assessing the overall magnitude of the association with recent mammography screening utilization. We acknowledge that there are variations in the strategies for measuring each of our exposures, which will require us to stratify our findings to better account for study heterogeneity.

For the analysis of the association between comorbidity and screening mammography utilization, we will separate studies that measure specific conditions from those evaluating comorbidity using a summary score. Reviewing both individual conditions and comorbidity indices will enable a comprehensive characterization of the most debilitating conditions that could affect screening mammography utilization. Since these are the primary methods of comorbidity measurement, stratified analyses will account for potential sources of heterogeneity.

When analyzing the association between functional limitations and screening mammography utilization, we will group studies that only use ADLs versus IADLs versus both ADLs and IADLs. Finally, for studies analyzing the association between health status and screening mammography utilization, we will separate studies that use a Likert scale health status measure from those using a prognostic index. Within studies using a prognostic index, we will perform subgroup analyses to compare studies that do and do not incorporate ADL or IADL measurements.

The primary outcome in our meta-analysis will be pooled odds ratios for screening utilization with corresponding 95 % confidence intervals. In our analyses, we will assess heterogeneity of studies to determine how structurally different studies are from each other. We will measure study heterogeneity using *I* ^2^ results and Cochran’s Q from the meta-analysis groupings. If there is no significant heterogeneity within our meta-analysis groupings based on *I* ^2^ results and Cochran’s Q, we will use the pooled results over the unpooled findings. While we anticipate that our studies have similar sample populations with similar conditions, we will use random-effects modeling to analyze study outcomes because it is a more conservative method to account for inherent variations between our studies under the assumption that the effects being estimated in included studies that are not identical.

Meta-regression controlling for study type, functional limitation, and comorbidity will be used to identify causes of heterogeneity. Our response variable will be the odds ratio of mammography utilization within the last 1–5 years. Along with study type and exposure differences (measures of functional limitation and comorbidity), we will also consider the study year and the minimum age of the study participants as potential covariates. We will account for potential residual heterogeneity and extra variability in our models by using random-effects modeling of our meta-regression.

We will also perform sensitivity analyses to examine potential publication bias including funnel plots, Begg’s test, Egger’s test, trim and fill, and jackknife analyses and report these findings in addition to the primary study findings and subgroup analyses [25]. Moreover, given the differences between the designs that could lead to different findings, we plan to perform a sensitivity analysis that separates randomized clinical trial from observational study findings. The meta-analysis results will also be graphically displayed using forest plots [25]. All analyses will be performed using STATA 13 (Stata, College Station, TX, USA).

#### Role of the study sponsor

The work represents collaboration between the authors, who represent a group of academic researchers at the University of California, San Francisco. All authors participated in the development of the study design, manuscript preparation, and the decision to submit the protocol for publication. All authors will participate in the data collection and analysis and will be responsible for data completeness and accuracy. The authors intend to publish the results for publication.

## Discussion

Because of heterogeneity in comorbidity, functional status, and life expectancy among older women, there is an urgent need for a more individualized approach to screening mammography in the elderly [19, 26–28]. The continuing controversy over screening mammography in older women indicates a need for evidence to guide informed choices [29]. Specifically, the consequences of screening older women have not been well described, especially in relation to life expectancy. Randomized trials of screening mammography cannot provide the evidence because the trials have excluded women older than age 74 and those with significant comorbidity [27]. This proposal will move the field forward by evaluating screening mammography utilization in older women across the levels of advancing age, comorbid illness, and functional status.

To our knowledge, this review will be the first to illuminate the impact of life expectancy on screening mammography uptake in older women. This evidence synthesis is particularly critical in women ages 65 years and older, a population that has both a higher incidence of breast cancer and also an increased risk of death due to competing causes. Generating direct and previously unavailable evidence will facilitate informed decisions regarding breast cancer screening policy for older women.

A related systematic review conducted by our group has examined the harms and benefits of breast cancer screening in older women in relation to comorbidity [19]. We extend this prior research by evaluating the role of comorbidity, functional status, and life expectancy in screening utilization. Moreover, Myers et al. recently reported that evidence of a relationship between screening and life expectancy, or quality-adjusted life expectancy, was lacking [30].

As life expectancy increases and screening mammography becomes more widely available, it is important to develop more individualized screening mammography approaches or life-expectancy-based screening [26]. For example, as of 2013, the Affordable Care Act requires Medicare to cover annual screening mammography at no cost to women starting at age 45 years with no upper age limit [31], which may lead to an increase in the rates of potentially inappropriate screening. Our proposed review will provide much needed evidence that will inform breast cancer screening policy and clinical decision aids.

### Reporting and dissemination of findings

We plan to report this review in accordance with the PRISMA guidelines [32] and to publish the findings in a peer-reviewed journal. We also intend to present these findings at relevant national and international scientific meetings.

## Appendix

### Search strategy

MEDLINE search:

*Search for Mammography Screening + Aged + Comorbidity*

(((("Breast Neoplasms"[Mesh]) AND (("Mammography"[Mesh]) OR "Mass Screening"[Mesh]))) AND ((("Aged"[Mesh]) OR ("Aged, 80 and over"[Mesh])) OR "Frail Elderly"[Mesh])) AND ((((((((((((((("Cardiovascular Diseases"[Mesh]) OR "Cognition Disorders"[Mesh]) OR "Comorbidity"[Mesh]) OR "Depression"[Mesh]) OR "Diabetes Mellitus"[Mesh]) OR "Disabled Persons"[Mesh]) OR "Hypertension"[Mesh]) OR "Kidney Diseases"[Mesh]) OR "Myocardial Infarction"[Mesh]) OR "Mental Disorders"[Mesh]) OR "Heart Diseases"[Mesh]) OR "Stroke"[Mesh]) OR "Intellectual Disability"[Mesh])) OR "Morbidity"[Mesh])

## Additional files

Additional file 1: Table S1. PRISMA-P (Preferred Reporting Items for Systematic review and Meta-Analysis Protocols) checklist: recommended items to address in a systematic review protocol. (DOC 53 kb)
Additional file 2: Table S2. Critical evaluation of the quality and limitations of the studies evaluating benefits and harms of screening mammography according to comorbidity. (DOC 42 kb)
Additional file 3: Table S3. Summary of findings from studies that evaluated the effect of summary measures of comorbidity burden on screening utilization. (DOC 40 kb)

## Abbreviations

ACS: American Cancer Society
ADLs: Activities of daily living
CINAHL: Cumulative Index to Nursing and Allied Health Literature
DB: Author 3
EMBASE: Excerpta Medica Database
IADLs: Instrumental activities of daily living
JD: Author 1
MeSH: Medical Subject Headings
PRISMA-P: Preferred Reporting Items for Systematic Review and Meta-Analysis Protocols
US: United States
USPSTF: United States Preventive Services Task Force
