# Supplementary material for: Utilization of screening mammography in older women according to comorbidity and age: protocol for a systematic review
Source: Syst Rev. 2016 Oct 4;5:168. doi: 10.1186/s13643-016-0345-y (PMC5050609; doi:10.1186/s13643-016-0345-y)
Supplement: Additional file 2: Table S2. — Critical evaluation of the quality and limitations of the studies evaluating benefits and harms of screening mammography according to comorbidity. (DOC 42 kb) [file 13643_2016_345_MOESM2_ESM.doc]

| **Additional file 2: Table S2.** Critical evaluation of the quality and limitations of the studies evaluating benefits and harms of screening mammography according to comorbidity | | | | | | | | | | | |
| --- | --- | --- | --- | --- | --- | --- | --- | --- | --- | --- | --- |
|  | Study Design | Selection | | | |  | Comparability of outcome groups | Outcome | | Evidence Quality^2^ |  |
| Source |  | Sample Representativeness | Sample Size | Non-Respondent Comparability | Exposure Ascertainment |  |  | Assessment | Statistical Test |  |  |
|  |  |  |  |  |  |  |  |  |  |  |  |
|  |  |  |  |  |  |  |  |  |  |  |  |
|  |  |  |  |  |  |  |  |  |  |  |  |
|  |  |  |  |  |  |  |  |  |  |  |  |
|  |  |  |  |  |  |  |  |  |  |  |  |
|  |  |  |  |  |  |  |  |  |  |  |  |
|  |  |  |  |  |  |  |  |  |  |  |  |
|  |  |  |  |  |  |  |  |  |  |  |  |
|  |  |  |  |  |  |  |  |  |  |  |  |
|  |  |  |  |  |  |  |  |  |  |  |  |
|  |  |  |  |  |  |  |  |  |  |  |  |
