# Supplementary material for: Utilization of screening mammography in older women according to comorbidity and age: protocol for a systematic review
Source: Syst Rev. 2016 Oct 4;5:168. doi: 10.1186/s13643-016-0345-y (PMC5050609; doi:10.1186/s13643-016-0345-y)
Supplement: Additional file 3: Table S3. — Summary of findings from studies that evaluated the effect of summary measures of comorbidity burden on screening utilization. (DOC 40 kb) [file 13643_2016_345_MOESM3_ESM.doc]

| **Additional file 3: Table S3.** Summary of findings from studies that evaluated the effect of **summary measures of comorbidity burden** on screening utilization | | | | | | |  |
| --- | --- | --- | --- | --- | --- | --- | --- |
| **Measures of comorbidity** | | **Source** | **Age** | **Measure of Association** |  |  |  |
|  |  |  |
| **Charlson Comorbidity Score** | |  |  |  |  |  |  |
|  |  |  |  |  |  |  |  |
|  |  |  |  |  |  |  |  |
|  |  |  |  |  |  |  |  |
|  |  |  |  |  |  |  |  |
|  |  |  |  |  |  |  |  |
| **Number of Comorbid Conditions** | |  |  |  |  |  |  |
|  |  |  |  |  |  |  |  |
|  |  |  |  |  |  |  |  |
|  |  |  |  |  |  |  |  |
|  |  |  |  |  |  |  |  |
|  |  |  |  |  |  |  |  |
|  |  |  |  |  |  |  |  |
